# Comparison of Six Different Silicones In Vitro for Application as Glaucoma Drainage Device

**DOI:** 10.3390/ma11030341

**Published:** 2018-02-27

**Authors:** Claudia Windhövel, Lisa Harder, Jan-Peter Bach, Michael Teske, Niels Grabow, Thomas Eickner, Ulf Hinze, Boris Chichkov, Ingo Nolte

**Affiliations:** 1 Small Animal Clinic, University of Veterinary Medicine Hannover, Foundation, D-30559 Hannover, Germany; claudia.windhoevel@tiho-hannover.de (C.W.); lisa.harder@tiho-hannover.de (L.H.); jan-peter.bach@tiho-hannover.de (J.-P.B.); 2 Institute for Biomedical Engineering, Rostock University Medical Center, D-18119 Rostock, Germany; michael.teske@uni-rostock.de (M.T.); niels.grabow@uni-rostock.de (N.G.); thomas.eickner@uni-rostock.de (T.E.); 3 Laser Zentrum Hannover e.V., D-30419 Hannover, Germany; u.hinze@lzh.de (U.H.); chichkov@iqo.uni-hannover.de (B.C.); 4 Leibniz Universität Hannover, D-30167 Hannover, Germany

**Keywords:** silicone, glaucoma, fibrosis, Med-4234, SILUPRAN 2445A/B, Silastic MDX4-4210, LSR 40, 40,026, Silbione LSR 4330, Silbione LSR 4350

## Abstract

Silicones are widely used in medical applications. In ophthalmology, glaucoma drainage devices are utilized if conservative therapies are not applicable or have failed. Long-term success of these devices is limited by failure to control intraocular pressure due to fibrous encapsulation. Therefore, different medical approved silicones were tested in vitro for cell adhesion, cell proliferation and viability of human Sclera (hSF) and human Tenon fibroblasts (hTF). The silicones were analysed also depending on the sample preparation according to the manufacturer’s instructions. The surface quality was characterized with environmental scanning electron microscope (ESEM) and water contact angle measurements. All silicones showed homogeneous smooth and hydrophobic surfaces. Cell adhesion was significantly reduced on all silicones compared to the negative control. Proliferation index and cell viability were not influenced much. For development of a new glaucoma drainage device, the silicones Silbione LSR 4330 and Silbione LSR 4350, in this study, with low cell counts for hTF and low proliferation indices for hSF, and silicone Silastic MDX4-4210, with low cell counts for hSF and low proliferation indices for hTF, have shown the best results in vitro. Due to the high cell adhesion shown on Silicone LSR 40, 40,026, this material is unsuitable.

## 1. Introduction

Silicones are widely used in medical applications, such as extracorporeal equipment in kidney dialyses, contact lenses, finger and foot joints, catheters, drains, shunts, breast implants, tubing, heart valves, ophthalmological implants [1], and as drug delivery systems [2]. They are often described as biocompatible, since they are chemically inert, stable against thermal and oxidative influences, and hypoallergenic [3,4]. Nevertheless, biocompatibility depends on the specified use and function of the implant [5]. The methyl groups on the siloxane polymer backbone cause hydrophobicity of silicone [6]. It is well known that wettability influences the cell adhesion of materials, and biocompatibility relating thereto [7,8]. This antiadhesive property is discussed as being a problem of the biocompatibility of breast implants [9], but is required by other implants [10].

In patients with glaucoma, insufficient drainage of aqueous humor, or uncontrolled production of aqueous humor, leads to a pathological increase in intraocular pressure (IOP) [11,12]. This increased IOP is a major cause of blindness worldwide [13]. If conservative therapies are not applicable or fail, glaucoma drainage implants are used to drain the water from the anterior chamber in the subconjunctival space to lower the pressure [14,15]. Often, the long-term success of these devices is limited by failure to control IOP due to fibrous encapsulation of the implant [16]. New designs and materials were developed to improve the clinical outcome [17,18,19,20], but biocompatibility still remains a problem, although implants made of silicone showed positive results in fibrosis and inflammatory reactions [19,20,21]. For this reason, this study focused on silicones as material for glaucoma drainage implants. 

Selection criteria were the potential to be used inside the human body for at least 29 days and manufacturer’s application notes, availability in laboratory scale, material properties (e.g., viscosity) that allow manual mixing, and a shore A hardness between 30–50 (which is needed for the mechanical function of the implant). According to these restrictions, the chosen and suitable silicones were NuSil, Med-4234 (material A); Wacker Chemie, SILPURAN 2445 A/B (material B); Dow Corning, Silastic MDX4-4210 (material C); Applied Silicone, LSR 40, 40026 (material D); Bluestar Silicones, Silbione LSR 4330 (material E) and Bluestar Silicones, Silbione LSR 4350 (material F). To the knowledge of the authors, there is no information on the use and suitability of these silicones as material for a glaucoma implant.

In the present study, an in vitro model was used to identify the cell-repellent characteristics of the previously named silicones as biomaterial for a new glaucoma drainage device. The aim was to identify the silicone with the best antiadhesive properties, under the presented manufacture method, to prevent fibrosis. Depending on the outflow area of the implant, different fibroblasts are responsible for excessive fibrosis. Most frequently, the external drainage part ends in the subconjunctival space [22]. In this area, episcleral fibroblasts, like human Tenon fibroblasts (hTF) and human Sclera fibroblasts (hSF), with a well characterized fibrotic potential, may cause encapsulation [23,24]. Both cell types are interesting for in vitro studies of new glaucoma drainage device materials. On the one hand, Tenon fibroblasts are used as a target for antiproliferative drugs intended to reduce fibrotic reactions in response to glaucoma implant surgery [25,26]. On the other hand, Sclera fibroblasts are interesting due to their participation in immunomodulatory processes [27] and their contribution to the plasticity of the eyeball with collagen remodelling [28]. Therefore, human Sclera fibroblasts and human Tenon fibroblasts cell lines were applied to analyze the influence of six different silicone types on cell proliferation, cell viability, and cell morphology on eyes. The aim was to identify the silicone with the best antiadhesive properties under the presented manufacture method.

## 2. Results

### 2.1. Silicones

**Figure 1:**
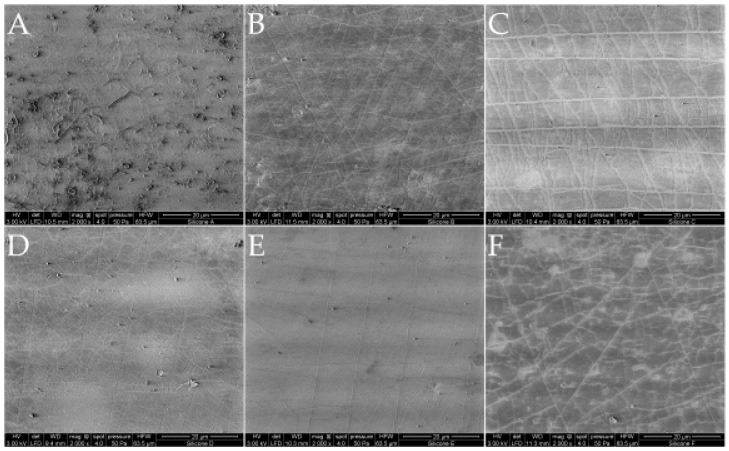
ESEM (environmental scanning electron microscopy) images Quanta FEG 250 (FEI, Eindhoven, The Netherlands) of the six silicones (material (**A**–**F**)). One film of each silicone was imaged with a 2000-fold magnification. Bars represent 20 µm.

The selected untreated silicone surfaces appeared homogenous and smooth, and showed no surface failure due to the manufacture process (Figure 1).

The water contact angle of the silicones was greater than 100 degrees on each silicone, and therefore, the surfaces were hydrophobic (Table 1). 

### 2.2. Cell Identity

To verify that the cultivated cells derived from fibroblasts, immunocytochemical fluorescence microscopy was performed. 

**Figure 2:**
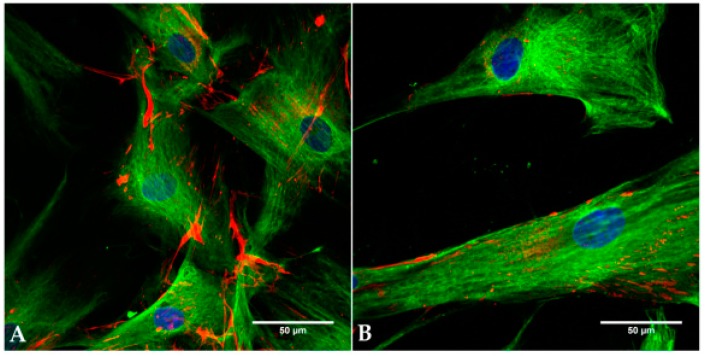
Immunocytochemical staining of hTF and hSF in vitro. After paraformaldehyde fixation and permeabilization with 0.2% Triton X-100, the cells were blocked with 1% bovine serum albumin (BSA). The cells were incubated with primary antibodies directed to the cytoskeleton (vimentin) and the compounds of ECM (fibronectin). Secondary antibodies were used to stain vimentin green (Alexa Fluor 488) and fibronectin red (Alexa Fluor 647). Nuclei were stained by DAPI (blue). HSF (P155) (**A**) and hTF (P3) (**B**) were positive for both markers. Bars represent 50 µm.

Vimentin, the major structural component of the cytoskeleton of mesenchymal origin-like fibroblast, could be detected in each fibroblast population (Figure 2). It was organized homogeneously intracellularly, and the cells showed a spindle-shaped morphology typical for fibroblasts.

Fibronectin is a protein of the extracellular matrix (ECM) network important for adhesion and cell–cell connections. This was detected in both cell lines (Figure 2). In an overlay with vimentin, it appeared to be located extracellularly. 

**Figure 3:**
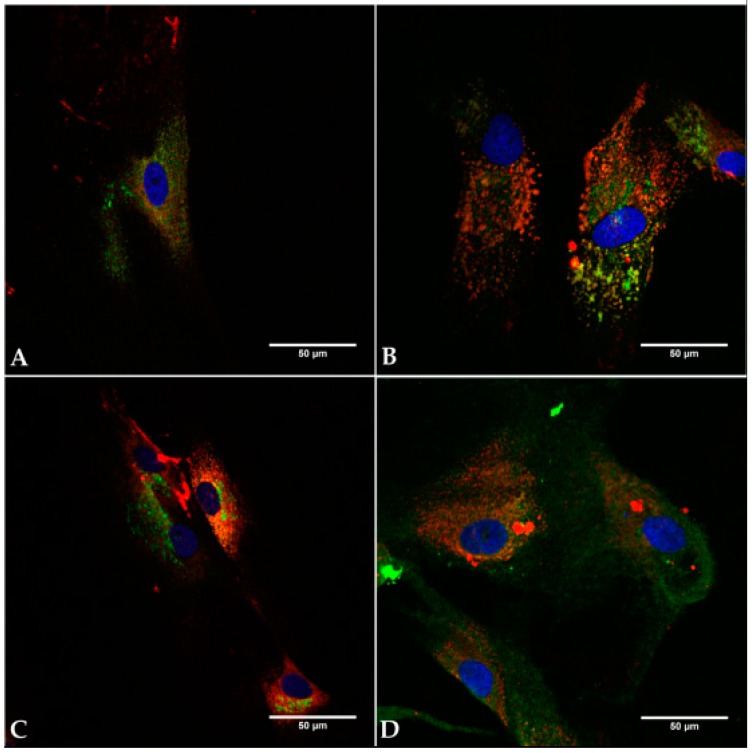
Demonstration of collagen I, III, and VI in hSF and hTF in vitro. After paraformaldehyde fixation and permeabilisation with 0.2% Triton X 100 the cells were blocked with 1% BSA. The cells were incubated with primary antibodies directed to the collagen I, III, and VI compounds of extracellular matrix (ECM). Secondary antibodies were used to stain collagen I, III green (Alexa Fluor 488) and collagen VI red (Alexa Fluor 647). Nuclei were stained by DAPI (blue). HSF (P155) (**A**,**C**) and hTF (P3) (**B**,**D**) showed intracellularly located collagen I and VI (**A**,**B**), and collagen III and VI (**C**,**D**). Bars represent 50 µm.

The capability to synthesize proteins of the collagen family characterizes the fibrotic potential of fibroblasts. The stained proteins collagen I, III, and VI, were only found perinuclearly, being intracellularly localized in both cell lines (Figure 3). As proteins of the ECM they were not organized extracellularly in fibrils or fibers. 

### 2.3. Cell Count

**Figure 4:**
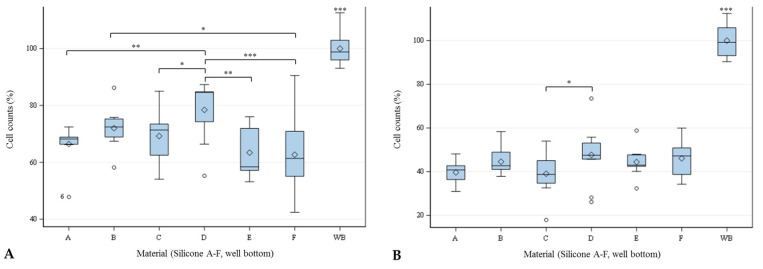
Cell counts in percent of hSF (**A**) and hTF (**B**) settled on different silicones (A–F) and well-plate bottom (WB) after 48 h. Global *F*-Test from the analyses of variance, followed by pairwise multiple means comparison with least significant difference test showed differences between the cell counts (* = *p* ≤ 0.05; ** = *p* ≤ 0.01; *** = *p* ≤ 0.001; *n* = 9; ◊ = mean; ─ = median; ◦ = outlier).

The cell counts were significantly affected by the silicones. Cell density of both cell lines was significantly lower on all silicones in comparison to the well bottom. As shown in Figure 4, hSF achieved the lowest cell density on silicone F (62.68% ± 13.97), and the highest density on silicone D (78.45% ± 11.07), compared to the well bottom (100% ± 5.82). HTF achieved the lowest cell density on silicone C (39.08% ± 10.53), and the highest density on silicone D (47.69% ± 14.26), compared to the well bottom (100% ± 7.39). 

### 2.4. Proliferation

The proliferation index was calculated for both cell lines using CFSE staining. The proliferation index describes the number of cell divisions divided by the number of cells that went into division.

**Figure 5:**
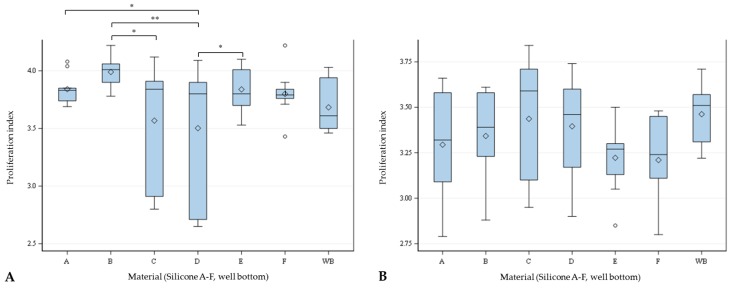
Proliferation indices of hSF (**A**) and hTF (**B**) settled on different silicones (A–F) and well-plate bottom (WB) after 48 h. Global *F*-Test from the analyses of variance, followed by pairwise multiple means comparison with least significant difference test showed differences between the cell counts (* = *p* ≤ 0.05; ** = *p* ≤ 0.01, *n* = 9; ◊ = mean; ─ = median; ◦ = outlier).

HSF growing on silicone C showed a significantly lower proliferation index compared with silicone B. Also, hSF on silicone D showed a significantly lower proliferation index compared with the indices on silicone A, B, and E. HTF showed similar proliferation behavior on the different materials. Differences between the materials are shown in Figure 5.

### 2.5. Viability

The cell viability represents the percentage of non-staining cells of the total cell count of each sample. HSF showed significantly higher viability growing on silicone F and WB than on silicones C, D, and E after 48 h. There was no statistically significant difference in the viability of hTF after 48 h. All means of the viability ranged between 83.14% and 91.76%, as shown in Table 2.

### 2.6. Live Cell Imaging (LCI)

**Figure 6:**
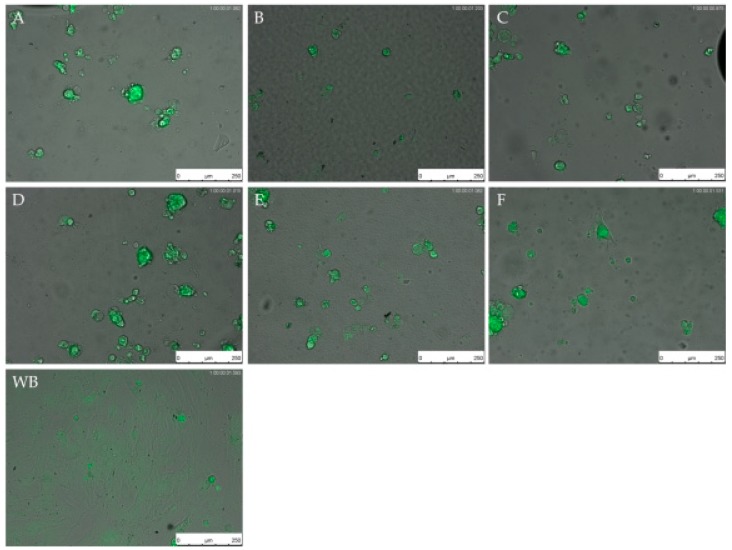
Live cell imaging (LCI) of CMFDA-stained human sclera fibroblasts (hSF) on different silicones (**A**–**F**) and well-plate bottom (**WB**). Images were taken at 100-fold magnification after 48 h. Bars represent 250 µm.

Images obtained with the LCI microscope showed pronounced differences in the morphology of the fibroblasts on silicones compared to the well-plate bottom (negative. control) (Figure 6). Cells on the well bottom showed a flattening of the cell body and a typical fibroblast morphology after a time. After 24 h, a confluent cell layer could be observed for both cell lines on the well bottom. The cells on the silicones were spherical over 72 h. Only some cells showed a flattening of the cell body, but no spreading like the cells on the well bottom. The cells on the silicones appeared in clusters. On the basis of these observations, the cell number was not determinable. 

## 3. Discussion

Biocompatibility refers to the ability of a material to cause appropriate host responses in a specific application [5]. Due to a wide variety of applications, requirements for biocompatibility differ. For example, a firm integration of orthopedic implants into the bone is desired for good stabilization [29], while stents remain isolated, in order to not become blocked by proteins or cells [30]. Again, other materials should dissolve over time (absorbable suture material) [31]. Therefore, choosing the appropriate biomaterial for a specific implantation site and function is an important step in the development of new implants prior to the outcome of animal testing or clinical trials.

Due to fibrous encapsulation of glaucoma drainage devices and the associated implant failure, a careful selection of the implant material is important [16,32,33]. Especially in this context, an implant material is required, which makes attaching of cell growth difficult.

Therefore, the topic of this study was to investigate different silicones for their eye fibroblast-repellent properties in vitro. The cell behavior of hSFs and hTFs on different silicones was compared, focusing on their attachment, proliferation, and viability. The authors searched for a silicone with very high antiadhesive properties, good processability, and a specific shore hardness between 30–50, to prevent postoperative fibrosis and encapsulation of potential glaucoma drainage implants. 

Cell adhesion is a complex process that depends on a variety of factors. Living cells cannot interact directly with synthetic materials, but over surface-bound proteins [7,34]. The binding of proteins to the surface determines the biocompatibility of the material. One of the most important proteins of the integrin family is fibronectin, responsible for adhesion of fibroblasts to the materials [35]. In the present study, fibronectin was detected extracellularly in both cell lines. Also, the collagens I, II, and VI were immunochemically detected perinuclearly after 48 h. These collagens are typically found in the ECM in fibrillar structures, which influence the adhesion, proliferation, and migration of cells. They are an important and crucial component of fibrous encapsulation [33,36,37]. The cells used in this study were able to express these proteins, and also adhered to the well bottom. However, a reduced protein adsorption due to the wettability of silicone is described [35]. The water contact angle measurements for all six silicones in this study were greater than 100°, and hence, the materials show a hydrophobic character. Several studies claimed the best water contact angle for cell adhesion to be between 55–70° [38,39]. This hydrophobicity explains the low cell counts on the silicones after 48 h, with a lower attachment of the cells on the surface. Silicone D showed the greatest cell count for both cell lines, and also had the lowest water contact angle (100 ± 9). The other silicones had higher measured water contact angles and lower cell counts. This observation was confirmed by the recording in the LCI. The cells on the well bottom showed a flattening of the cell body even after a short contact with the bottom, and thus, an attachment to the substrate. In the course of the observations, an increase in the overgrown area was observed. By contrast, cells of both cell lines showed a spherical morphology on the silicones over the entire period. The cells attached to the silicones appeared smaller than the cells on the well bottom. Many cells were arranged in grape-like clusters, which is explained by the stronger cell–cell cohesion on hydrophobic surfaces than the cell–substrate binding [40]. 

Only silicones were tested in this study, because comprehensive in vivo testing has already shown their benefits, although no silicones are mentioned by the product name in literature. Ayyala et al. [19,20] inserted different materials (polypropylene, silicone, Vivithane, Acrosof intraocular lens) into the subconjunctival space in rabbits, and evaluated outcomes both clinically and histologically. Silicone was reported to cause mild to moderate fibrosis and few inflammatory reactions. Lower attachment of fibroblasts was observed on silicone than on polypropylene, Vivithane, and intraocular lens material after explanation of the materials. This is underlined by clinical outcomes of the Ahmed Glaucoma Drainage Device model FP7, consisting of silicone, and model S2 of polypropylene [21,41,42]. The silicone implant shows lower IOP, fewer complications, and a better success rate. Since neither the manufacturer nor the composition of the silicones of glaucoma drainage devices are described in the literature, silicones were chosen for screening that have a good processability and possess the physical properties for a new glaucoma implant. ESEM images during the manufacturing process showed no quality defects in the form of cracks or trapped air.

Similar results of the viability between the silicones and the well bottom were expected, due to the preselection of medically approved silicones. Before use in humans, all new materials must be tested for biocompatibility and toxicity in accordance with DIN EN ISO 10993–5. In this study, neither hSF nor hTF with direct contact to the material showed increased cell death after a cultivation time of 48 h (cut-off point 70%). The hSF showed less viability than hTF, and therefore appeared to be more sensitive to the silicone materials. The cell-repelling properties should therefore be directed primarily against hTF, as these are the starting tissue of the fibrous capsule [24]. It can be assumed that a higher cell death will occur over a longer period of time, due to the lower adhesion to the silicone surface [43]. 

When proliferation, viability, and cell count are considered together, there is no silicone achieving the best result in every characteristic. To prevent a new intraocular pressure increase due to blocking of the drainage, low cell attachment is important. Therefore, the silicones E and F, with low cell counts for hTF and low proliferation indices for hSF, and silicone C, with low cell counts for hSF and low proliferation indices for hTF, have shown the best results, in vitro, in the present study. Due to the high cell adhesion on silicone D in comparison to the other silicones, this material is unsuitable. The higher amount of cells on the material may lead to a faster implant failure. 

In addition to the intended properties in cell culture, the processability is also important. As the silicones C, E, and F showed problems in processing, only silicones A and B are suitable for the preparation of a new implant, due to their processability and their results in the in vitro test.

Physical (such as surface irregularities, roughness, shape, flexibility) and chemical properties (such as wettability [40]) of a material affect the binding of proteins, and hence, the attachment and growth of cells [44,45,46,47,48,49,50]. Choritz et al. [51] analyzed the surface topographies of glaucoma drainage devices and their influence on human Tenon’s fibroblast adhesion in vitro, and discussed the roughness of the material as one important aspect leading to the differences in cell attachment.

In this study, similar surface roughness for all silicone samples are assumed, due to the identical manufacturing process. ESEM images are used to control the manufacturing, and showed no quality defects in the form of cracks or trapped air.

Limitations of the study are the complex processes in the eye that cannot be demonstrated in the cell culture. Cell-lined fibrous capsules can be found around the tested biomaterials in eyes which were histologically observed, but no cells were attached to the silicone after explantation [19,20]. In addition, previous studies discuss the influence of cytokine and growth factors in the aqueous humor on the foreign body reaction [32,52]. Although the material of a new implant was tested in vitro in the present study, in vivo tests are necessary to prove implant function. 

In conclusion, it can be said that the silicones are biocompatible materials which are suitable for the production of glaucoma drainage implants, due to their cell-repellent properties. The reduced binding of proteins to the surface lowers adhesion and proliferation of fibroblasts on the surface significantly. However, the viability of cells remains good. Although the results of the cell culture experiments show no great differences, it is possible to enable a pre-selection of a material, especially in accordance with the manufacturing process for new glaucoma drainage devices.

The in vitro model is an inexpensive, reproducible, well-established and safe method to obtain valuable information about the cell behavior on different materials, and to preselect materials before starting experiments in vivo.

## 4. Materials and Methods

### 4.1. Silicone

The materials were prepared in accordance with the suppliers’ instructions and data sheets. For liquid materials, a glass substrate (75 × 25 mm^2^) was prepared with spacers of well-defined height (e.g., 140 µm) at the margins. The material was poured into the center, covered with another glass substrate, and cured according to the supplier instructions. For viscous materials, more robust polymethyl methacrylate (PMMA) substrates were prepared with spacers at the margin. The material was applied in the center, covered with another PMMA substrate, compressed, and cured in accordance with the supplier’s instructions. After curing, the substrates were carefully released to obtain the silicone film. 

Material A, NuSil, Med-4234 (NuSil Technology LLC, Carpinteria, CA, USA) is a two-part, translucent, pourable silicone elastomer. It consists of two components (part A and part B, viscosity 86 Pa s and 440 mPa s) that have to be mixed at a ratio of 10:1. The pourable material was cured with heat via addition-cure chemistry. Specified hardness Shore A is 30. The manufacturer states that it may be considered for use in human implantation for a period of greater than 29 days. 

Material B, Wacker Chemie, SILPURAN 2445 A/B (Wacker Chemie AG, Munich, Germany) is a two-part addition-curing RTV-2 silicone rubber curing to a silicone elastomer of medium hardness. It is pourable after stirring. It consists of two components (part A and part B, viscosity 10 Pa s) that have to be mixed at a ratio of 1:1. After curing for 5 min at 177 °C, the material is translucent. Specified hardness Shore A is 40. Pot life is 10 min. Films could be easily removed from substrates. The material is intended for use in prosthetics; biocompatibility in accordance with USP Class VI is stated.

Material C, Dow Corning, Silastic MDX4-4210 (Dow Corning GmbH, Midland, MI, USA) is a two-part silicone elastomer. It consists of two components (viscosity 115 Pa s) that have to be mixed at a ratio of 10:1. Curing of the pourable material is achieved in about 3 days at room temperature. Specified hardness Shore A is 30. Films that we processed from this soft material had a tendency to wrinkle and tear. The material is designed for medical device components and qualified for 30 days of physical contact in accordance with ISO 10993-1 tests. 

Material D, Applied Silicone, LSR 40, 40026—10:1 Implant Grade (Applied Silicone Corporation, Santa Paula, CA, USA) is a two-part liquid silicone rubber that cures to a rubbery elastomer via addition-cure chemistry. It consists of two components A and B (viscosity 150 Pa s) that have to be mixed at a ratio of 1:1. Curing is achieved in 4 h at 50 °C. Specified hardness Shore A is 40. Films could be easily removed from substrates. The material has been approved by per ISO 10,993 for long term implantation.

Material E, Bluestar Silicones, Silbione LSR 4330 and Material F, Bluestar Silicones, Silbione LSR 4350 (Bluestar Silicones, West Point, GA, USA) are two-part liquid silicones. They consist of two components that are mixed in equal parts (viscosity 550 Pa s and 2000 Pa s). Curing was achieved in 15 s at 175 °C; no post cure was needed. The material is available in various levels of hardness Shore A from 1 to 70; Silbione 4330 comes with a specified hardness of 30, Silbione 4350 with a hardness of 50. Applications are general molded parts and medical applications for <29 day implantation. When manually mixing, air inclusions could occur in the viscous material. Prepared as a thin film, Silbione LSR 4330 is less tear-resistant than Silbione LSR 4350.

#### 4.1.1. Characterization of the Silicones

##### Environmental Scanning Electron Microscope (ESEM)

The six different silicones were analyzed by ESEM Quanta FEG 250 (FEI, Eindhoven, The Netherlands) to control defects in the surface, such as cracks, air bubbles, deposits, or cavities caused by the manufacturing process. Both sides of one film of each silicone were imaged with a 40-fold, 500-fold, 1000-fold, and 2000-fold magnification, respectively.

##### Water Contact Angle

To evaluate the wetting characteristics, water contact angle measurements were performed using a video-based optical contact angle measuring system (OCA 20 Micro, DataPhysics Instruments GmbH, Filderstadt, Germany), and were calculated by the Young–Laplace method [53]. The static contact angles for water were analyzed by sessile drop method, with a volume of 4 µL per drop with a flow rate of 0.5 µL/ s at room temperature (20 °C) and normal atmospheric pressure. The baseline was set manually, the contour of the drop was evaluated automatically, and the angle was calculated by the corresponding software SCA 20 (version 3.12.10, DataPhysics Instruments GmbH, Filderstadt, Germany). Ten measurements of each silicone were taken.

#### 4.1.2. Preparation of the Silicone for In Vitro Analysis

The prepared silicones were sterilized, maintaining a temperature of 121 °C for at least 15 min by using saturated steam under at least 210 kPa pressure. Thin samples with a diameter of 6 millimeters were punched from the silicone films for in vitro testing. 

### 4.2. Cell Culture

#### 4.2.1. Human Sclera Fibroblast (hSF)

The sclera fibroblasts were prepared from donor eyeballs (Institute of Anatomy, Rostock University Medical Center, Rostock, Germany) as previously described [22]. Fibroblasts were cultured in 25 cm^2^ and 75 cm^2^ cell culture flasks (TPP, Faust Lab Science, Klettgau, Germany), and incubated at 37 °C in 5% CO_2_ humidified air. The cell culture medium, Dulbecco’s Modified Eagle Medium low glucose (DMEM) (Sigma-Aldrich, Munich, Germany) containing 10% fetal calf serum (FCS) (Biochrom GmbH, Berlin, Germany) and 1% penicillin and streptomycin (pen/strep) (Biochrom GmbH, Berlin, Germany), was changed twice a week, and cells were cultivated until reaching confluency.

#### 4.2.2. Human Tenon’s Fibroblast (hTF)

Small samples of approximately 1 × 2 mm^2^ of Tenon’s capsule were obtained during routine glaucoma surgery from donors who had given prior consent, following the declaration of Helsinki. The hTFs were stored in a sterile tube in phosphate-buffered saline (PBS) (Biochrom GmbH, Berlin, Germany) at room temperature until being processed. The different storage solutions did not influence further cultivation. Cells were rinsed with PBS and transferred to a 40 mm cell culture dish (TPP, Faust Lab Science, Klettgau, Germany). Using forceps, the hTFs were spread, applying light pressure on them. After 1 min, 500 µL of complete cell culture medium (DMEM low glucose/10% FCS/1% pen/strep) were added. The hTFs were incubated at 37 °C with 5% CO_2_ in humidified air. On the following day, 1.5 mL cell culture medium was filled in slowly. The hTFs were monitored on a daily basis, and medium was changed three times a week. Tissues were placed in a 25 cm^2^ culture flask using forceps when cells had grown out of the sample, which took one to two weeks. The cells were passaged into the same flask, using TrypLE™ Express (Life Technologies GmbH, Darmstadt, Germany). Afterwards, cells were routinely cultured in 75 cm^2^ cell culture flasks. 

#### 4.2.3. Immunocytochemical Staining

The cell origin was confirmed by immunocytochemical staining for vimentin, fibronectin, and collagen I, III, and VI. HSF passage 155 (P155) and hTF (P3) were seeded in a µ-Slide 8-well plate (ibidi GmbH, Munich, Germany) with a concentration of 2500 cells per well. After 48 h of cultivation, the cells were fixed using 4% paraformaldehyde buffered with PBS for 20 min at room temperature. For intracellular staining, the fixed cells were permeabilized using 0.2% Triton X-100 (Sigma-Aldrich Chemie GmbH, Munich, Germany) in PBS for 20 min at room temperature. After blocking with 1% bovine serum albumin (BSA) (Sigma-Aldrich Chemie GmbH, Munich, Germany) for 30 min, the cells were incubated with primary antibodies (Abcam, Cambridge, UK). These were used in the following dilutions: rabbit monoclonal anti-fibronectin 1/1000 (ab32419), mouse monoclonal anti-vimentin 1/200 (ab8069), mouse monoclonal anti-collagen I 1/100 (ab6308), mouse monoclonal anti-collagen III 1/100 (ab6310), and rabbit monoclonal anti-collagen VI 1/100 (ab6588). After washing with PBS, the cells were incubated with secondary antibodies: goat polyclonal anti-rabbit IgG H+L (Alexa Fluor 647) 1/250 (Cell Signaling Technology, Leiden, The Netherlands) and goat anti-mouse IgG H+L (Alexa Fluor 488) 1/1000 (ab150117) for 1 h at room temperature, and subsequently rinsed again with PBS. Unspecific staining of antibodies was excluded from the control experiments by using only the secondary antibodies. Nuclei were stained by 4,6-diamidino-2-phenylindole 1/1000 (DAPI) (Sigma-Aldrich Chemie GmbH, Munich, Germany). Fluorescent labelling was studied using a confocal fluorescence microscope (Eclipse TE2000-E, Nikon, Düsseldorf, Germany) with a 60× water immersion objective (Nikon). Images were taken using EZ-C1 1.80 software (Nikon).

### 4.3. Characterization of Cell Behaviour on Silicones

#### 4.3.1. Cell Counting

The influence of six different silicones on Sclera and Tenon fibroblast growth was evaluated by cell count. First, 96-well cell culture plates (Corning, New York, NY, USA) were prepared with 36 sterilized circular films of each silicone for each cell line. Purpose-built Teflon rings (external radius 3.3 mm, inner radius 2.1 mm) were used to fix the silicone at the well bottom. Teflon rings were also inserted into 36 wells without silicone films, as untreated well-plate bottom control (TC-treated polystyrene). Cells, hSF (P160) and hTF (P6), were seeded at a concentration of 5000 cells per well. After incubation for 48 h, the adherent cells were washed with PBS and trypsinized with 150 µL TrypLE™ Express. To collect all cells, each well was washed with 150 µL cell culture medium and were merged to a uniform cell suspension. Suspensions from four wells were pooled to finally obtain nine samples for each silicone, and for the reference from well-plate bottom. The pooled cell suspensions were analyzed using flow cytometry in MACSQuant^®^ Analyser 10 (Miltenyi Biotech GmbH, Gladbach, Germany). The cells were gated in FlowJo 7.6.1 software (FloJo, LLC, Ashland, OR, USA) using forward scatter (FSC) and side scatter (SSC), to include only single, viable cells, and to eliminate any debris, dead cells, and clumps or doublets. The mean of numbers of cells achieved from the untreated well-plate bottom was set at 100%, in order to achieve a better comparability between the experiments.

#### 4.3.2. Staining of hSF and hTF for Cell Division Tracking and Viability

HSF (P155) and hTF (P6) were used for cell tracking division with CellTrace^TM^ CFSE Cell Proliferation Kit (Life Technologies GmbH, Darmstadt, Germany). Cells (2 × 10^6^) were prepared and labelled with carboxyfluorescein succinimidyl ester (CFSE) at a concentration of 5 µM. After 20 min incubation at 37 °C in a 5% CO_2_ atmosphere, staining was stopped using 10 mL ice-cold culture medium. After 5 min, cell suspension was rinsed twice using cell culture medium. Cell culture plates (96-well) were prepared with silicone films in accordance with 2.3.1. Twelve wells with 10 × 10^4^ stained cells per silicone and the well-plate bottom (WB) were cultured. Four wells with unstained cells were cultured as reference for auto fluorescence. Stained cells (25 × 10^4^ per well) were seeded in four wells of a second plate prepared with 150 µL ChillProtec©-Medium (Biochrom AG, Berlin, Germany), and were stored at 4 °C, keeping the cells in the starting condition (generation zero). After 48 h, the cells were washed with PBS, trypsinized with 150 µL TrypLE™ Express, and collected as described in 2.3.1. Four wells were pooled and centrifuged for 10 min at 1000 rpm (Hettich UNIVERSAL 320 R, Tuttlingen, Germany) at room temperature. The cell pellet obtained was resuspended in 200 µL TO-PRO^®^-3 Iodide 1/1000 (Life Technologies GmbH, Darmstadt, Germany) for live/dead staining. The cell suspensions were analyzed using flow cytometry in FACSCalibur (BD Biosciences, Heidelberg, Germany). Data analysis was performed using FlowJo 7.6.1 software. The experiment was performed in triplicate.

#### 4.3.3. Live Cell Imaging (LCI) of hSF and hTF

LCI of cells on implant materials was performed as recently described [54]. Cell culture plates (96-well) were prepared for imaging. Four sterilized films of each silicone were placed on the well-plate bottom and fixed using Teflon rings. Teflon rings were also inserted into four wells without silicone films as untreated well-plate bottom control. Culture medium (150 µL) was filled into every well. HSF (P156) and hTF (P5) were stained with CellTracker™ Green CMFDA (5-chlormethylfluoresceindiacetat) (Life Technologies GmbH, Darmstadt, Germany) in accordance with the manufacturer’s instructions. The cells were gently rinsed with PBS, and stained with 25 µM CMFDA for 30 min. After staining, the cells were detached using TrypLE^TM^ Express and 5 × 10^3^ cells were seeded into each prepared well. The plates were placed in the LCI Microscope (DMI 6000B Leica Microsystems, Wetzlar, Germany) at 37 °C and 5% CO_2_ for 72 h. Every 30 min, an image was taken of three defined positions of every well using the program LAS AF 2.6.0 at a magnification of 100. The overlay images were used for further analysis.

### 4.4. Statistical Analysis

Statistical analysis was performed using SAS^®^ software 7.1 (SAS Institute Inc., Cary, NC, USA). Cell number, viability, and proliferation index were evaluated using the global *F*-test from the one-way analysis of variance followed by pairwise multiple means comparisons with the least significant difference test. *p*-values < 0.05 were considered to be statistically significant. 

## Figures and Tables

**Table 1:** Water contact angle measurements on silicones (A–F) (*n* = 10).

Silicone	Water Contact Angle Mean ± SD [°]
A	119 ± 7
B	117 ± 3
C	122 ± 4
D	100 ± 9
E	103 ± 9
F	118 ± 10

**Table 2:** Viability (%) of hSF and hTF after TO-PRO®-3 Iodide. Staining on silicones (A–F) and well-plate bottom (WB) after 48 h (*n* = 3). The mean value of the cell number on the WB was set at 100%.

Material		A	B	C	D	E	F	WB
**Viability (%)**	**hSF mean ± SD**	87.46 ± 5.10	87.76 ± 3.98	84.07 ± 9.84	83.14 ± 4.19	84.48 ± 9.84	90.43 ± 3.10	90.08 ± 3.51
	**hTF mean ± SD**	91.76 ± 2.72	90.03 ± 6.62	92.50 ± 1.92	91.21 ± 2.58	91.21 ± 2.56	90.96 ± 1.91	91.61 ± 1.63
